# Tough Talks COVID-19 Digital Health Intervention for Vaccine Hesitancy Among Black Young Adults: Protocol for a Hybrid Type 1 Effectiveness Implementation Randomized Controlled Trial

**DOI:** 10.2196/41240

**Published:** 2023-02-13

**Authors:** Henna Budhwani, Allysha C Maragh-Bass, Elizabeth E Tolley, Maria Leonora G Comello, Marie C D Stoner, Margo Adams Larsen, Donald Brambilla, Kathryn E Muessig, Audrey Pettifor, Christyenne L Bond, Christina Toval, Lisa B Hightow-Weidman

**Affiliations:** 1 Intervention Research and Implementation Science Lab College of Nursing Florida State University Tallahassee, FL United States; 2 Behavioral, Epidemiological, Clinical Sciences Division FHI360 Durham, NC United States; 3 Hussman School of Journalism and Media University of North Carolina at Chapel Hill Chapel Hill, NC United States; 4 RTI International Research Triangle Park, NC United States; 5 Virtually Better, Inc Decatur, GA United States; 6 Institute on Digital Health and Innovation College of Nursing Florida State University Tallahassee, FL United States; 7 Gillings School of Global Public Health University of North Carolina at Chapel Hill North Carolina, NC United States

**Keywords:** COVID-19, COVID, vaccine hesitancy framework, African American, young adults, implementation science, digital health, mHealth, behavioral intervention, vaccination, intervention, mortality, USA

## Abstract

**Background:**

Interventions for increasing the uptake of COVID-19 vaccination among Black young adults are central to ending the pandemic. Black young adults experience harms from structural forces, such as racism and stigma, that reduce receptivity to traditional public health messaging due to skepticism and distrust. As such, Black young adults continue to represent a priority population on which to focus efforts for promoting COVID-19 vaccine uptake.

**Objective:**

In aims 1 and 2, the Tough Talks digital health intervention for HIV disclosure will be adapted to address COVID-19 vaccine hesitancy and tailored to the experiences of Black young adults in the southern United States (Tough Talks for COVID-19). In aim 3, the newly adapted Tough Talks for COVID-19 digital health intervention will be tested across the following three southern states: Alabama, Georgia, and North Carolina.

**Methods:**

Our innovative digital health intervention study will include qualitative and quantitative assessments. A unique combination of methodological techniques, including web-based surveys, choose-your-own-adventures, digital storytelling, user acceptability testing, and community-based participatory approaches, will culminate in a 2-arm hybrid type 1 effectiveness implementation randomized controlled trial, wherein participants will be randomized to the Tough Talks for COVID-19 intervention arm or a standard-of-care control condition (N=360). Logistic regression will be used to determine the effect of the treatment arm on the probability of vaccination uptake (primary COVID-19 vaccine series or recommended boosters). Concurrently, the inner and outer contexts of implementation will be ascertained and catalogued to inform future scale-up. Florida State University’s institutional review board approved the study (STUDY00003617).

**Results:**

Our study was funded at the end of April 2021. Aim 1 data collection concluded in early 2022. The entire study is expected to conclude in January 2025.

**Conclusions:**

If effective, our digital health intervention will be poised for broad, rapid dissemination to reduce COVID-19 mortality among unvaccinated Black young adults in the southern United States. Our findings will have the potential to inform efforts that seek to address medical mistrust through participatory approaches. The lessons learned from the conduct of our study could be instrumental in improving health care engagement among Black young adults for several critical areas that disproportionately harm this community, such as tobacco control and diabetes prevention.

**Trial Registration:**

ClinicalTrials.gov NCT05490329; https://clinicaltrials.gov/ct2/show/NCT05490329

**International Registered Report Identifier (IRRID):**

DERR1-10.2196/41240

## Introduction

### Background

Interventions for increasing the uptake of COVID-19 vaccination among young adults are central to ending the pandemic [1]. Given their high rate of asymptomatic infection [2], young adults represent a priority population on which to focus efforts for promoting COVID-19 vaccine uptake [1]. The acceptance of COVID-19 vaccination is lower among African American or Black (henceforth referred to as *Black*) young adults aged 18 to 29 years. In 2020, a population-based study indicated that only 42% of Black Americans reported being likely to accept vaccination against COVID-19, whereas 63% of White and Hispanic or Latinx adults were likely to accept COVID-19 vaccination [3]. In a nationally representative survey on the same topic, lower vaccine acceptance was associated with younger age [4]. Although the vaccination gap between the Black and White adult populations has narrowed, the disparity between Black and White young adults aged 18 to 29 years persists, with Black young adults being at about a 14% disadvantage when compared to their White peers [5]. COVID-19 has exacerbated the disparities experienced by Black young adults, particularly those residing in the southern United States, where health care access continues to be a barrier, and the vaccination gap between White and Black southern US adults is about 7.2% [5,6]. From May to August 2020, Black individuals accounted for 18.7% of COVID-19 deaths despite making up just 12.5% of the US population [7]. Stigma, discrimination, and distress are highest among those with intersectional identities (eg, Black, young, rural, etc) [8], reinforcing health inequities [9,10]. Recent studies found that respondents who reported experiences of racial discrimination had increased odds of higher vaccine hesitancy when compared to those who did not report such experiences and that vaccine hesitancy was intertwined with institutional distrust [11,12].

Over the past year, there have been notable changes in the epidemiology, public health recommendations, and pandemic-related mitigation strategies surrounding COVID-19. The rates of COVID-19 vaccination are currently at 61.8% in Alabama, 64.3% in Georgia, and 82.2% in North Carolina, and these rates continue to be lower among Black populations in these southern states [13]; yet, only 34.1%, 34.9%, and 25.1% of Alabama, Georgia, and North Carolina residents, respectively, have accepted a booster. Those who remain unvaccinated may be highly resistant toward accepting vaccination or overwhelmingly apathetic due to the numerous, fast-paced changes in COVID-19 knowledge and messaging that occurred during the course of the past year.

Digital health interventions (DHIs) can reach young adults regardless of geographic location and stigmatizing experiences with health care institutions [14]. DHIs can enable young adults to make informed decisions about their health, using a familiar modality that young adults value and trust. DHIs are well suited for young adults, given the ubiquity of technology use. A recent report found that 96% of 18- to 29-year-old adults in the United States owned a smartphone [15]. As such, smartphones are suitable for delivering content that is tailored to each user’s unique needs. Further, DHIs have been shown to increase knowledge, self-efficacy, and motivation for change while also ameliorating distrust, fear, and stigma across a variety of health conditions [16-21]. Young adults already rely on digital technologies to build their social networks, receive social support, and obtain health information [18,22,23]. Access to credible web-based resources is critical, given that the majority of young adults access COVID-19 information from web-based news and social media sites [24].

Considering these factors, we developed this protocol to adapt a previously developed DHI in the domain of HIV status disclosure (Tough Talks) to a COVID-19 vaccine decision-making context (Tough Talks for COVID-19 [TT-C]) [25]. The TT-C intervention aims to enable Black young adults in the southern United States to make informed, autonomous decisions about COVID-19 vaccine receipt via nonstigmatizing and tailored messaging. Through engaging activities and narrative communication, TT-C will address the structural contexts (eg, issues of confidence, distrust in medicine, and stigma), misinformation (eg, vaccination knowledge), environmental barriers (eg, access to care and health insurance), and potential consequences (eg, outcomes related to accepting or refusing vaccination) [26] related to vaccine decisions. Using community-based participatory research (CBPR) methods to cocreate TT-C with Black young adults will promote personal agency, bolster resilience in the face of the pandemic, strengthen intervention quality, and increase intervention relevance and engagement [27-29].

### The CBPR Approach

CBPR is a collaborative approach to science that involves authentic partnerships between researchers and the community being supported [30]. The goals of CBPR are to collaboratively create solutions, such as an intervention (as is the case in our study), that are meaningful and helpful to the community and to partner with the community to address potential concerns or barriers. CBPR acknowledges that the community has expertise and that active participation and community feedback are essential to project success [30,31]. Considering the persistent mistreatment of Black communities within health care institutions that has resulted in skepticism and distrust toward new health interventions and research studies, the study team felt that it was imperative to adopt CBPR within the TT-C DHI research project.

### Framework

**Figure 1:**
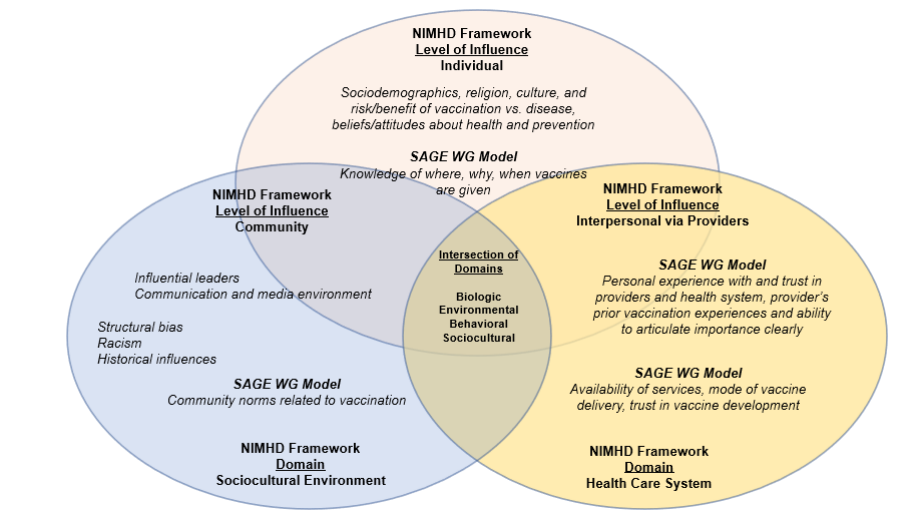
Vaccine hesitancy model for US-based minoritized populations. The model is informed by the NIMHD research framework and the World Health Organization Strategic Advisory Group of Experts. NIMHD: National Institute of Minority Health Disparities. SAGE WG: Strategic Advisory Group of Experts Working Group.

Vaccine hesitancy is influenced by factors at the individual, community, provider, health care system, and societal levels [32]. As espoused by the World Health Organization Strategic Advisory Group of Experts, individual and social group influences, contextual influences, and vaccine-specific issues must be identified and targeted through multicomponent and tailored interventions to increase vaccine uptake within key populations [33]. We will therefore utilize the National Institutes of Health/National Institute of Minority Health Disparities research framework to assess multilevel factors at the individual (knowledge, attitudes, and normative beliefs), interpersonal (peer and family influences), institutional (provider communication and health care system), and structural levels (stigma and discrimination) that inﬂuence COVID-19 vaccine hesitancy and refusal. We have created a model (informed by the Strategic Advisory Group of Experts Working Group; Figure 1) that depicts the determinants that are relevant to understanding and addressing vaccine hesitancy among Black young adults [34]. This model will be used to guide the development of our intervention.

### Objectives

Our protocol includes 3 objectives (ie, specific aims). For aim 1, we conducted multimethod formative research to elicit the behavioral, cognitive, and environmental determinants influencing COVID-19 vaccine hesitancy among Black young adults. For aim 2, in collaboration with expert advisors, community partners, and Black young adult end users, we will leverage the ADAPT-ITT (assessment, decision, administration, production, topical experts, integration, training, and testing) framework [35] to develop and refine TT-C. For aim 3, we will conduct a hybrid type 1 effectiveness implementation randomized controlled trial (RCT) with 360 Black young adults from Alabama, Georgia, and North Carolina to assess the effectiveness of TT-C in increasing COVID-19 vaccine uptake while concurrently evaluating the implementation contexts that affect intervention delivery.

### Timeline

Ours is a 4-year (48-month) study, and regulatory activities will be conducted between months 1 and 6 of the study. The development team will work to adapt the Tough Talks platform to create TT-C during months 6 to 18, which will include the internal testing of the platform’s features and usability (aims 1 and 2). Following feedback from the full investigative team, our expert advisors, the community, and youth advisory members, the intervention will be tested for feasibility and acceptability with youth through a technical pilot during months 18 to 24 (aim 2). We plan to launch the RCT by month 24, which will conclude at month 48 (ie, at the end of 4 years; aim 3).

## Methods

### Ethics Approval

All study materials and procedures were reviewed and approved by the University of North Carolina, Chapel Hill Institutional Review Board (approval number: 21-1746) and by the Florida State University Institutional Review Board (STUDY00003617). Informed consent will be collected digitally from all study participants prior to data collection and randomization. Study data will be deidentified and stored on a secure server. Study participants will receive an incentive of a US $50 gift card payment for each data collection.

### Young Adult Advisory Board

Young adults from 3 states will be recruited for the TT-C young adult advisory board (YAB) through our community and academic partners, and we will pay special attention to ensuring diversity in age, gender, and vaccine-related perceptions. The TT-C YAB will be formed in the first quarter of the study and will meet monthly for the entire project period. The TT-C YAB will provide guidance on (1) young adults’ knowledge, beliefs, and attitudes toward COVID-19 vaccination; (2) sources of vaccine misinformation and disinformation, support, and resource needs; and (3) strategies for communicating with Black young adults to address vaccine hesitancy. The health inequities laid bare by the pandemic and the calls for social justice for racial minorities will be the center of this approach. We will ensure that TT-C YAB members are supported to share their lived experiences and advise the investigator team on addressing racism and discrimination in TT-C.

### Community Advisory Group

We include respected experts in health disparities, social justice and equity, CBPR, community activism and advocacy, and stigma. Our advisory board includes scientists of color, leadership from historically Black colleges and universities, and community advocates. Members will provide guidance on all aspects of the study. The full board will meet biannually; individual board members will meet with the study team as needed to provide feedback.

### Recruitment

For all aims, our recruitment strategy includes (1) free and paid advertising and posting on social media sites; (2) information distribution through national organizations working with Black young adults, including historically Black colleges and universities; and (3) information distribution through our community partners and other network collaborations. We will use methods that were successfully deployed in our prior studies to recruit participants through social media sites that are frequented by Black young adults and rely on our network of community and academic partners for additional dissemination [36,37]. The recruitment procedures may vary slightly depending on the community organization and study phase, but all recruitment materials will use consistent messaging that frames the study as a mechanism for learning more about COVID-19 vaccines.

### Eligibility Criteria

The aim 1 eligibility criteria include being aged 18 to 29 years; identifying as Black; being proficient in English; having access to a personal smartphone; and being a current resident of Alabama, Georgia, or North Carolina. The aim 2 eligibility criteria include being aged 18 to 29 years; identifying as Black; being proficient in English; having access to a personal smartphone; being a current resident of Alabama, Georgia, or North Carolina; and being hesitant toward COVID-19 vaccines. The aim 3 eligibility criteria include being aged 18 to 29 years; identifying as Black; being proficient in English; having access to a personal smartphone; being a current resident of Alabama, Georgia, or North Carolina; and being out of compliance with vaccination guidelines [38]. Participants who do not comply with vaccination guidelines include both individuals who have never received a COVID-19 vaccine and those who have not received any booster.

### Screening for Eligibility

All potential participants (whether recruited via the internet or in person) will complete a web-based screening survey via Qualtrics (Qualtrics International Inc) to provide consent for screening and verify all inclusion criteria. For those who met the eligibility criteria for aim 1, the screening survey directed participants to an informed consent video with accompanying text. Those who meet eligibility criteria for aims 2 and 3 will be asked to record their first name, email address, and phone number if they are interested in participating in the study. Those who are disinterested in participating can decline by exiting the website. Potential participants who do not meet the eligibility criteria will be asked if they would like to be contacted about other research studies and, if so, to provide contact information.

### Aim 1

#### Overview

At the time of publication, data collection for aim 1 has already been completed. Aim 1 included the following two distinct phases: a web-based survey and digital storytelling workshops. A total of 150 Black young adults were recruited from Alabama, Georgia, and North Carolina to participate in a web-based survey. A subsample was invited to participate in a web-based digital storytelling workshop to produce digital stories that will become part of the TT-C intervention.

#### Aim 1 Survey

Participants completed a one-time web-based survey through the Qualtrics survey platform, which included both validated survey constructs and choose-your-own-adventure formats [39,40]. Within the choose-your-own-adventure portion, participants were presented with culturally and contextually realistic scenarios, and we asked them to make decisions and reflect on the outcomes of these decision. Each story path included multiple branch points where participants made a behavioral choice. Each choice impacted the subsequent information presented and later scenario branches. Short multiple-choice questions and open-ended questions were included within each path to assess how participants made their choices and to capture their reflections on these decisions. This survey took about 30 to 45 minutes to complete. Participants who reported high levels of vaccine confidence were invited to participate in the digital storytelling workshop.

#### Aim 1 Digital Storytelling Workshop

Interested and eligible survey respondents were directed to informational text that described the digital storytelling workshop and its purpose [41]. Once the participants consented, they were invited to participate in 1 of 2 web-based digital storytelling workshops; each consisted of 3 sessions. Each workshop session lasted about 3 hours, and sessions were conducted 1 week apart. Participants were informed that the goal was to develop a video that would be included in an intervention that aims to help peers learn from their COVID-19 pandemic–related lived experiences. During the workshops, participants created digital stories (1- to 3-minute videos) that included still and moving images, voice-over recordings of the participants telling their stories, and background music or text to document their experiences related to the COVID-19 pandemic and why they chose to be vaccinated. Select digital stories will be incorporated into a TT-C module entitled “Hear from you peers.” This module will be available to aim 3 participants who are randomized to the intervention condition.

### Aim 2

#### Overview

Up to 16 young adults who have expressed COVID-19 vaccine hesitancy will participate in web-based focus groups. The focus groups will consist of 2 rounds of usability testing activities that will be conducted 1 to 2 months apart. During round 1, participants will review TT-C content, generate additional ideas, and identify assets on which to build, guided by the ADAPT-ITT framework (Table 1) [35]. Young adults will provide recommendations and context-relevant insights regarding any requested additions or changes. The study team will collectively review the recommendations and prioritize and address the actionable ideas to be presented for further feedback and testing with YAB members. Beta testing will (1) allow for a full assessment of intervention content (eg, identification of any remaining spelling, text, or graphical errors) and technical functionality, (2) ensure that the back-end collection of app analytics data is being performed appropriately, and (3) help finalize pilot test procedures. All errors and technical bugs will be addressed or optimized prior to the RCT.

**Table 1:** ADAPT-ITT^a^ model: Phases and methodology.

ADAPT-ITT phase	Aim	Study method
Assessment	1	Formative research with Black young adults via a web-based survey, digital storytelling, and focus groups
Decision	2	Adapt Tough Talks based on established feasibility, acceptability, and early effectiveness data
Administration	2	Usability testing with Black young adults in Alabama, Georgia, and North Carolina
Production	2	Produce initial intervention prototype
Topical Experts	2	Review TT-C^b^ with identified scientific and community experts and young adult advisors
Integration	2	Integrate content from topical experts, young adult advisors, and Black young adult participants of the technical pilot
Training	2	Train staff on TT-C app functionality
Testing	3	Conduct hybrid type 1 effectiveness implementation randomized controlled trial

^a^ADAPT-ITT: assessment, decision, administration, production, topical experts, integration, training, and testing.

^b^TT-C: Tough Talks for COVID-19.

#### TT-C Intervention and Control Condition

The TT-C intervention will be finalized to be self-directed and fully asynchronous, so that there will be no need for the participants to engage with an interventionist. The initial assessments suggest that to complete all activities and review all content, the study participants will need to dedicate about 2 to 4 hours of time (spread across 1 month). Our standard-of-care control will be the provision of COVID-19 vaccine materials, which will be adapted from the Centers for Disease Control and Prevention and shared via email. Standard-of-care materials will be hosted via the internet and will be accessible to the general public. In the final phase of aim 2, the YAB and community partners will participate in an assessment of the TT-C intervention to assess intervention feasibility and acceptability and finalize RCT study procedures.

### Aim 3

#### Overview

In aim 3, we will conduct a hybrid type 1 effectiveness implementation RCT. We will enroll 360 Black young adults from Alabama, Georgia, and North Carolina who are unvaccinated, have only received 1 dose of a 2-dose series, or have not received boosters against COVID-19 in accordance with current Centers for Disease Control and Prevention booster eligibility criteria [42]. After collecting informed consent, using block randomization by state, we will allocate 180 participants to each arm. The web-based screener will automatically randomize participants (N=360) to the control condition (n=180) or the TT-C intervention arm (n=180). Self-report data, including vaccination status, will be collected at baseline and at 1 and 3 months after baseline.

#### Statistical Power

Power calculations were conducted based on the assumptions that dropout would be equivalent across arms and approximately 15% of participants would be lost to follow-up; therefore, we assume that 306 participants will complete the primary end point assessment. If the rate of COVID-19 vaccination in the control arm is between 10% and 20%, we will have 80% power to detect an odds ratio of 1.92 to 3.21 when comparing vaccination uptake between arms, assuming a sample size of 306 and a 2-sided α of .05. We anticipate that the vaccination rate in the control arm will be low, given that COVID-19 vaccines have been available for some time. Therefore, we expect that a total sample size of 360 will provide sufficient power to detect a clinically meaningful difference in vaccine uptake.

#### Aim 3 Effectiveness Measures and Analysis

The aim 3 primary effectiveness outcome is COVID-19 vaccine uptake, which will be defined as the receipt of any COVID-19 vaccine (primary series or boosters). The secondary effectiveness outcomes are vaccine hesitancy and confidence [43], as well as vaccine knowledge, attitudes, and beliefs [44]. We will also collect quantitative data on implementation outcomes, the social cognitive theory constructs targeted by TT-C, COVID-19–specific risks and behaviors, and pertinent constructs that have been validated in the extant literature on Black young adults (Table 2).

**Table 2:** Effectiveness measures.

	Baseline	Follow-up
Number and percentage of participants who receive any new vaccine during the study period (eg, participants who accept the first dose of a COVID-19 vaccine, complete the vaccine series, or receive a booster shot)		✓
Vaccine hesitancy [32], confidence [43], knowledge [45], attitudes, and beliefs [32]	✓	✓
Usability (including acceptability; ie, TT-C^a^ satisfaction and completion), demand (including the number of youths screened, enrolled, and retained), and practicality (based on the TT-C intervention’s cultural appropriateness and fit) [46]		✓
Physical environmental factors (access, utilization, and vaccine misinformation), social environmental factors (discrimination, as measured via the Adapted Everyday Discrimination Scale for Medical Settings [47]; stigma; social support, as measured via the MOS^b^ social support survey [48]; social norms; and medical mistrust, as measured via the Group-Based Medical Mistrust Scale [49]), outcome expectations (COVID-19–specific knowledge, attitudes, and beliefs), observational learning (vaccination [eg, flu or HPV^c^] experiences of self or others), and perceived self-efficacy (vaccination barriers and facilitators)	✓	✓
Sociodemographic characteristics (age, race, ethnicity, gender identity, income, employment, and education [eg, current student])	✓	✓
Pre-existing conditions (eg, diabetes, cardiovascular conditions, etc)	✓	✓

^a^TT-C: Tough Talks for COVID-19.

^b^MOS: Medical Outcomes Study.

^c^HPV: human papillomavirus.

We will use logistic regression to determine the effect of the treatment arm on the probability of vaccination uptake (“yes” or “no”) at month 3. We will focus on the odds ratios for comparing the probability of vaccination in the treatment group with the probability of vaccination in the control group. As an exploratory analysis, we will examine primary and secondary outcomes by enrollment vaccination status (not vaccinated vs vaccinated with 1 or 2 doses and no booster shot). The secondary outcomes—vaccine hesitancy [4]; vaccine confidence [43]; and vaccine knowledge, attitudes, and beliefs [44]—will all be assessed by using previously validated scale scores, of which each will consist of the continuous sum of responses to a series of questions with Likert scale responses. We will use linear regression models with treatment group as the exposure to determine whether continuous secondary outcomes vary among treatment groups. Lastly, we will use the causal mediation framework proposed by Valeri and Vanderweele [50] to understand the mechanisms for any intervention effects through each of our hypothesized mediators [50-53]. We will use the *g-formula* command in Stata (StataCorp LLC), which uses the mediational g-formula, to estimate the controlled direct effects, natural direct effects, and natural mediated (indirect) effects [54].

In sensitivity analyses, we will also use inverse probability of treatment weights to account for missing data and potential differential retention between study arms over the study period. We will weight outcome models by using stabilized inverse probability of censoring weights that include the treatment arm as a predictor to account for differential loss to follow-up.

#### Aim 3 Implementation Outcomes and Analysis

We will conduct qualitative interviews with a group of purposively selected participants (we estimate 18-24 participants) to assess how the intervention was internalized and experienced. We will conduct in-depth exit interviews on a rolling basis after participants have concluded the 3-month study cycle. All interviews will be audio-recorded and transcribed verbatim. Qualitative analyses will be conducted in the NVivo (QSR International) coding program. Study participants will be asked questions on how sociocultural nonintervention contexts affected the likelihood of vaccine acceptance, vaccine series completion, and vaccine hesitancy. Interviews will last about 1 hour. We will perform a rapid content analysis, which is routinely used in implementation science to assess and disseminate results quickly and accurately [55]. The findings will be explored as a function of participant age, sex, and state. Qualitative findings will provide direct insight into real-world implementation considerations.

## Results

Our study was funded at the end of April 2021. Approval from the University of North Carolina, Chapel Hill Institutional Review Board was obtained shortly thereafter. Aim 1 data collection concluded in early 2022. The entire study is expected to be complete at the end of January 2025.

## Discussion

### Overview

The TT-C intervention will address COVID-19–related vaccine hesitancy among Black young adults in Alabama, Georgia, and North Carolina by including tailored content to engage Black young adults and promote agency and autonomy in decision-making related to vaccination, while also addressing the persistent effects of historic contexts and structural stigma and racism against Black populations in the United States. The TT-C DHI will address issues that not only impact COVID-19 vaccine uptake but also influence the acceptance of other vaccines and health services, such as addressing medical distrust, highlighting the role of Black scientists in vaccine development and medicine, and providing tailored content on how to assess misinformation. The TT-C app will be developed to retain relevance as the pandemic shifts, and it can be adapted to address other conditions that disproportionately affect Black young adults.

### Limitations

In assessing trial designs, we considered stepped wedge and waitlist formats. However, since our aim is to reach Black young adults who are often not engaged in care, particularly those in the southern United States, we determined that multipronged recruitment and individual-level randomization would be the best approaches. Because the landscape of COVID-19 prevention and related science are quickly changing (eg, masking, the definition of *fully vaccinated*, etc), ideally, the app should be flexible and responsive. Although the content delivered through the TT-C app is adaptable, not all components can be adjusted once they are developed.

### Conclusion

If the TT-C DHI is effective in increasing COVID-19 vaccination uptake among Black young adults in the southern United States, we will be poised for rapid dissemination and broad implementation to reduce COVID-19 morbidity and mortality among unvaccinated or partially vaccinated Black young adults in the southern United States.

## Abbreviations

ADAPT-ITT: assessment, decision, administration, production, topical experts, integration, training, and testing
CBPR: community-based participatory research
DHI: digital health intervention
RCT: randomized controlled trial
TT-C: Tough Talks for COVID-19
YAB: Young Adult Advisory Board

## Data Availability

The data sets generated during aim 3 of our study will be shared in compliance with National Institutes of Health data sharing policies and will be made available from the senior author on reasonable request.
